# Neuronal population coding of perceived and memorized visual features in the lateral prefrontal cortex

**DOI:** 10.1038/ncomms15471

**Published:** 2017-06-01

**Authors:** Diego Mendoza-Halliday, Julio C. Martinez-Trujillo

**Affiliations:** 1 McGovern Institute for Brain Research, Department of Brain and Cognitive Sciences, Massachusetts Institute of Technology (MIT), Cambridge, Massachusetts 02139, USA; 2 Department of Physiology, McGill University, Montreal, Quebec, Canada H3G 1Y6; 3 Departments of Physiology, Pharmacology and Psychiatry, Robarts Research Institute, Brain and Mind Institute, Schulich School of Medicine and Dentistry, Western University, London, Ontario, Canada N6A 5B7

## Abstract

The primate lateral prefrontal cortex (LPFC) encodes visual stimulus features while they are perceived and while they are maintained in working memory. However, it remains unclear whether perceived and memorized features are encoded by the same or different neurons and population activity patterns. Here we record LPFC neuronal activity while monkeys perceive the motion direction of a stimulus that remains visually available, or memorize the direction if the stimulus disappears. We find neurons with a wide variety of combinations of coding strength for perceived and memorized directions: some neurons encode both to similar degrees while others preferentially or exclusively encode either one. Reading out the combined activity of all neurons, a machine-learning algorithm reliably decode the motion direction and determine whether it is perceived or memorized. Our results indicate that a functionally diverse population of LPFC neurons provides a substrate for discriminating between perceptual and mnemonic representations of visual features.

## Abstract (web summary)

Neurons in the lateral prefrontal cortex are known to encode visual features as well as maintain them in working memory. Here the authors report that LPFC neurons encode both perceived and memorized visual features in diverse combinations and the population activity reliably decodes as well as differentiates between these two representations.

Primates heavily rely on visual information to perform behavioural tasks. This information can be accessible in two main ways: in some instances, visual stimuli containing such information remain available to the eyes and are perceived during task execution; in other instances, the stimuli become unavailable and the brain must temporarily maintain and monitor a working memory (that is, internally-driven) representation of the task-relevant visual information. One important question is whether the same or different neural substrates encode visual information while it is perceptually available and while it is unavailable and maintained in working memory.

The primate lateral prefrontal cortex (LPFC) is thought to play a role in both accessing and monitoring perceptual representations^1^, as well as maintaining information in working memory^2^,^3^. Neurons in the LPFC of macaque monkeys encode the locations or features of stimuli that are perceptually available^1^,^4^. In addition, during the memory period of delayed match-to-sample tasks, LPFC neurons also encode the remembered location or non-spatial features of visual stimuli^2^,^5^,^6^,^7^. However, it remains unclear whether the same or different neurons within LPFC encode visual features while they are perceptually available (perceptual coding), and when they are held in working memory (mnemonic coding).

In the current study, we examined the ability of each LPFC neuron, as well as the population, to encode a non-spatial visual feature when it is perceived and when it is memorized. We recorded the activity of LPFC neurons in two macaque monkeys while they performed a task in which the relevant visual feature—motion direction—either remained perceptually available for the entire trial or became unavailable during the trial and had to be maintained in working memory. We found that neurons encoded perceived and memorized directions to various degrees and in different combinations of strength. Furthermore, using population decoding analyses, we show that the combined activity pattern of this diverse neuronal population was capable of reliably coding for both perceived and memorized directions, as well as distinguishing between them.

## Results

### Task and behavioural performance

**Figure 1:**
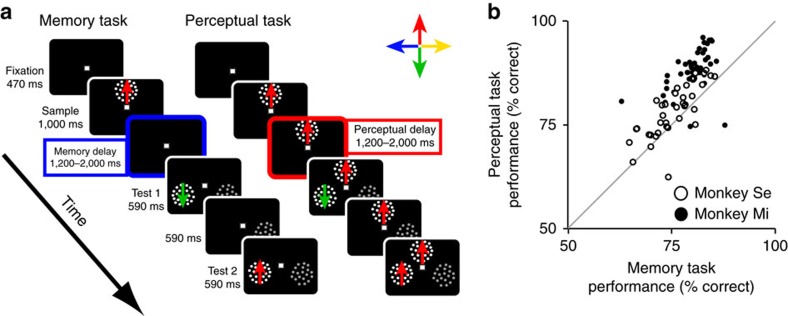
Behavioural task design and performance. (**a**) Temporal sequence of visual display during the two tasks. Trials of the two tasks were randomly interleaved. The motion directions of all random-dot stimuli are represented by colour-coded arrows. The duration of each display is indicated. The memory delay and perceptual delay periods are highlighted with blue and red, respectively. Stimuli are not drawn to scale. A stimulus with 0% coherent motion and lower luminance contrast (shown in grey) was presented simultaneously with the test stimuli on the opposite hemifield for purposes that are irrelevant to the present study. (**b**) Performance of each monkey in each recording session (circle) in the memory task (horizontal axis) and perceptual task (vertical axis). Unity line is shown in grey.

We trained two rhesus macaque monkeys to perform two variants of a match-to-sample task in which they viewed a moving random-dot stimulus (the sample), followed by two test stimuli sequentially presented at one of two possible locations. The animals had to report with a button release which of the two test stimuli had the same motion direction as the sample (Fig. 1a). In the memory task, the sample disappeared after one second, and after a delay period randomly varied between 1,200 and 2,000 ms (that is, memory delay), the test stimuli were presented. Hence, to assess the match between the sample and test directions, the monkeys were required to maintain the sample direction in working memory during the memory delay. The perceptual task was identical to the memory task except in that the sample remained on the display during the delay period (that is, perceptual delay; 1,200 to 2,000 ms long) and until the end of the trial; thus, the monkeys had continuous visual access to the sample direction during the perceptual delay and were not required to memorize it. Trials from the two tasks were randomly interleaved. Our study included data collected during 30 recording sessions from monkey Mi and 34 from monkey Se.

In both tasks and all sessions, the performance of both animals was considerably higher than chance (chance=50%; Fig. 1b), and significantly higher in the perceptual than the memory task (paired *t* test; Monkey Mi: *t*(41)=10.51, *P*<0.001; Monkey Se: *t*(39)=2.04, *P*=0.049), indicating that the continuous presence of the sample stimulus during the perceptual task conveyed an advantage to the monkeys with respect to the memory task.

### Coding of perceived and memorized directions by LPFC neurons

**Figure 2 f2:**
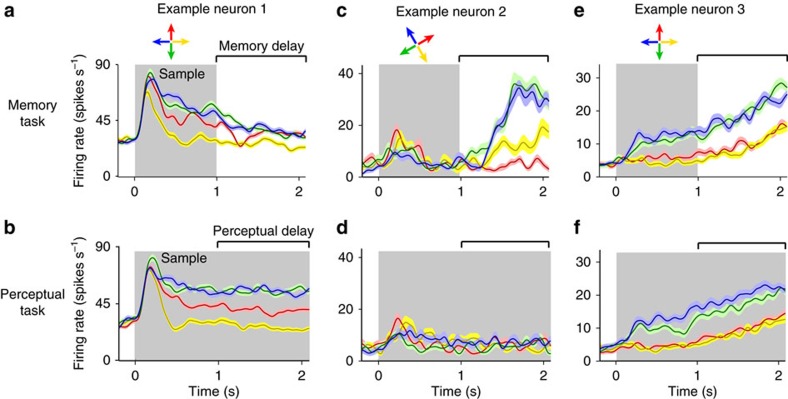
Diversity of LPFC neuronal responses during the perceptual and memory tasks. (**a**–**f**) Mean firing rate (±s.e.) over time in trials with each of the four sample directions (colour-coded arrows) for three example LPFC neurons during the memory task (**a**,**c**,**e**) and the perceptual task (**b**,**d**,**f**). Grey area, sample presentation period. Brackets, delay period.

We recorded the activity of 272 single LPFC neurons in two monkeys. Neurons were located in cortical sites surrounding the posterior segment of the principal sulcus [Fig f2][Fig f3][Fig f4][Fig f5][Fig f6][Fig f7](Fig. 8a), mainly within areas 8a and 9/46 (ref. 8). We first examined whether the activity of neurons encoded the sample direction during each of the two tasks. For example, the neuron in Fig. 2a,b encoded the sample direction more strongly during the perceptual than the memory task. In contrast, the second example neuron (Fig. 2c,d) encoded the sample direction during the memory delay but not during the perceptual delay. The third example neuron (Fig. 2e,f) similarly encoded the sample direction during both the perceptual and memory delay periods.

We quantified each neuron's ability to discriminate between directions (discriminability) using the area under the receiver operating characteristic curve (auROC) obtained by comparing the distribution of firing rates for the neuron's preferred and least-preferred directions (see Methods). Discriminability during the delay period of the perceptual and memory tasks was used as a measure of the strength of perceptual and mnemonic coding, respectively. Our results were quantitatively similar between animals (Supplementary Fig. 1), and are presented hereon as pooled data. In 126 of the 272 recorded neurons (46%), direction discriminability was significantly higher than expected by chance in at least one of the two tasks (permutation test; see Methods). Overall, 28% of all neurons showed significant perceptual discriminability, whereas 32% showed significant mnemonic discriminability.

**Figure 3 f3:**
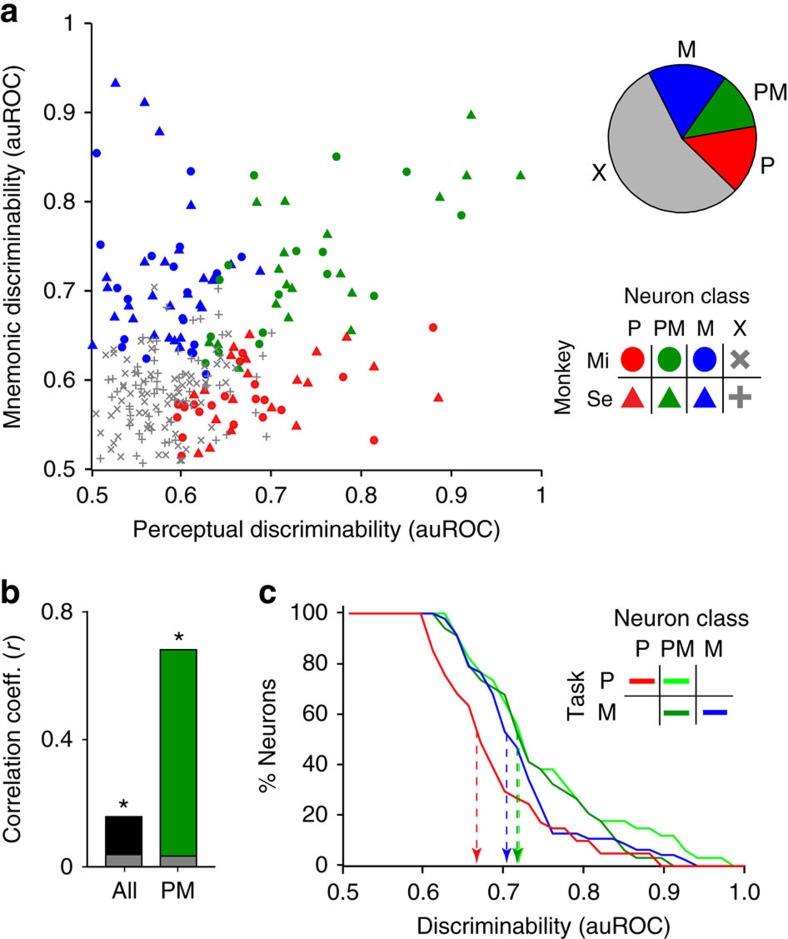
Perceptual and mnemonic discriminability of LPFC neurons. (**a**) Scatter plot showing perceptual (horizontal axis) and mnemonic (vertical axis) discriminability for each neuron recorded in each of the two monkeys (Mi and Se). Neurons are classified by the significance of discriminability (P, perceptual; PM, perceptual–mnemonic; M, mnemonic; X, no discriminability). Top-right, pie chart showing the proportion of neurons of each class. (**b**) Linear correlation coefficient (*r*) between perceptual and mnemonic discriminability for all neurons (black) and perceptual/mnemonic neurons (green). Asterisk, significant *r*; ns, non-significant *r*; grey bars, correlation expected by chance. (**c**) Reverse cumulative distribution of discriminability across the recorded neurons, separated by neuron class and task. Dashed lines, median auROC for each distribution.

We then considered several alternative scenarios: first, that perceived and memorized directions are encoded by the same LPFC neurons, with each neuron showing similar strength of perceptual and mnemonic coding; second, that the neurons coding for perceived directions are completely different from those coding for memorized directions; third, that different neurons encode perceived and memorized directions to different degrees, in such a way that the population contains a wide variety of combinations of coding strengths for perceived and memorized directions. We found many neurons with strong perceptual coding but weak mnemonic coding, and vice versa, thus discarding the first scenario. In addition, many neurons strongly encoded both perceived and memorized directions, thus discarding the second scenario. Our results were best described by the third scenario (Fig. 3a): neurons fell along a continuum of combinations of perceptual and mnemonic coding strength and showed no apparent clustering into separate functional categories. However, along that continuum, only a fraction of the neurons showed statistically significant perceptual and/or mnemonic discriminability. We therefore classified neurons as perceptual, mnemonic or perceptual–mnemonic based on this statistical significance. Of all neurons with delay period direction tuning, 32% were perceptual, 40% were mnemonic and 28% were perceptual–mnemonic (Fig. 3a). The percentage of perceptual–mnemonic neurons was significantly higher than expected if the probabilities of each neuron showing perceptual and mnemonic discriminability were independent of each other (permutation test, *P*=0.0007; see Methods). Very similar results were obtained by alternatively using the proportion of explained variance between the responses in the four sample direction conditions to assess each neuron's direction discriminability (Supplementary Fig. 2; see Methods).

The observed diversity of coding among neurons raised the question of whether there was any relationship between neurons' perceptual and mnemonic coding strength. We found a significant positive correlation between perceptual and mnemonic discriminability across all recorded neurons (Fig. 3b, *r*=0.16, *P*=0.013). In neurons with significant direction discriminability in both tasks (perceptual–mnemonic), this correlation was more than three times stronger, and also significantly above chance (Fig. 3b, *r*=0.68, *P*<0.00001). Such correlation was not merely induced by the method of neuronal classification, since the significance of perceptual and mnemonic discriminability were independently tested using data from different trials (see Methods). These results indicate that while there is a diverse combination of coding strength for perceived and memorized directions among all neurons, there is a positive relationship between the two types of coding. On the basis of this relationship, one may anticipate that perceptual–mnemonic neurons would have higher perceptual and mnemonic coding than perceptual and mnemonic neurons, respectively. In effect, perceptual–mnemonic neurons had significantly higher perceptual discriminability than perceptual neurons (Fig. 3c; *P*=0.002, Wilcoxon rank sum test), and higher mnemonic discriminability than in mnemonic neurons, although this difference did not reach significance (Fig. 3c; *P*=0.36, Wilcoxon rank sum test).

### Population decoding of perceived and memorized directions

Given the presence of neurons with preferential coding of perceived or memorized directions, we hypothesized that the activity pattern of the LPFC neuronal population should contain enough information to determine not only the sample direction but also the nature of the representation (that is, perceived or memorized). To test these predictions, we pooled all the recorded neurons to create a pseudopopulation, and their activity during the delay periods was used to train a machine-learning algorithm (linear discriminant analysis) to classify each trial as belonging to one of the eight experimental conditions (two tasks with four sample directions each) using a leave-one-out cross-validation method.

**Figure 4 f4:**
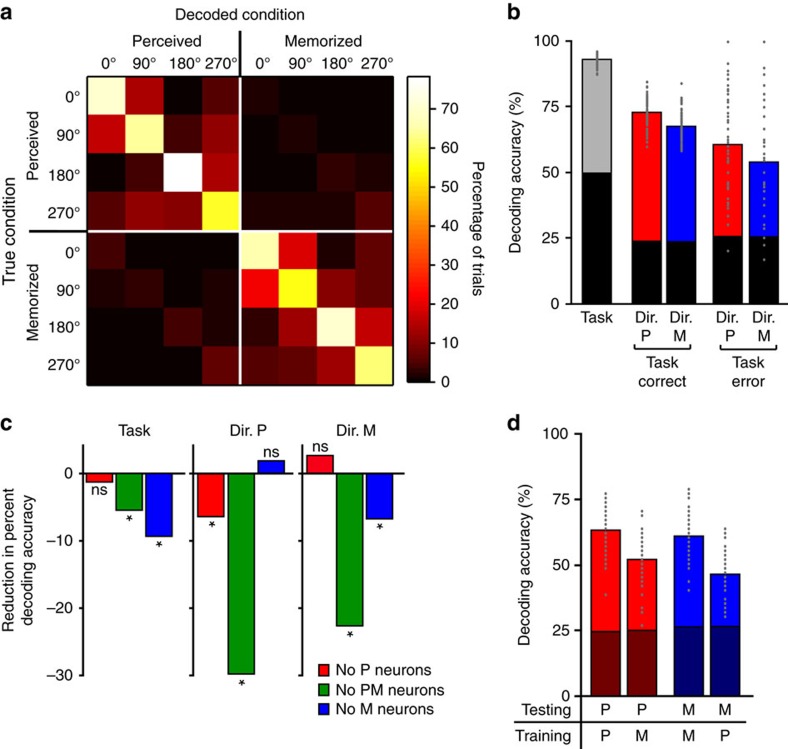
Population decoding of task and motion direction. (**a**) Confusion matrix showing the percentage of trials of a given condition (true condition) that were decoded as belonging to each of the eight conditions (decoded condition). Right, colour scale. (**b**) Percentage of trials classified as the correct task (grey) or the correct sample direction during the perceptual task (red) or memory task (blue). Direction decoding accuracy was separately measured in trials for which task was correctly or incorrectly decoded. (**c**) Reduction in decoding accuracy for task (left), perceived direction (centre) and memorized direction (right) caused by removal of neurons of each functional class (perceptual, red; perceptual–mnemonic, green; mnemonic, blue). Asterisk, significant reduction; ns, not significant. (**d**) Percentage of trials with correct direction decoding obtained by training with trials of the perceptual or memory task (P or M) and testing with trials of either task. In **b**,**d** dark-colored bars show chance decoding accuracy; grey dots, performance from individual bootstraps.

The confusion matrix in Fig. 4a shows the percentage of trials of a given condition that were classified into each of the eight conditions. The highest decoding incidences fell along the descending diagonal, showing that the decoder classified most trials into the correct conditions. Performing the same decoding analyses separately on neuronal populations from each individual monkey yielded similar results (Supplementary Fig. 3). We first measured the ability of the classifier to decode whether the sample direction was perceived or memorized (that is, task decoding), by computing the percentage of trials classified into a condition belonging to the correct task, regardless of direction. These trials are represented in the top left and bottom right quadrants (delineated by white lines) in the confusion matrix of Fig. 4a. Task decoding accuracy was 91%, far above the 49.9% value expected by chance (Fig. 4b; randomized trial labels test, *P*<0.001).

Among trials with correct task classification, we computed the percentage with correct sample direction classification. Direction decoding accuracy was significantly higher than chance in both tasks (Fig. 4b; randomized trial labels test, *P*<0.001 for both tasks), and was significantly higher in the perceptual task than the memory task (Fig. 4b, bootstrap, *P*<0.001), indicating that in LPFC, population coding is stronger for perceived than memorized directions. Importantly, the distribution of direction preferences among selective neurons was similar between the two tasks, and therefore did not influence the above result (Supplementary Fig. 4a). Given the close link between neuronal activity in LPFC and behaviour^9^, it is interesting that this result is consistent with the observation that the monkeys performed significantly better in the perceptual than the memory task (Fig. 1b).

To examine the differential contribution of perceptual, mnemonic and perceptual–mnemonic neurons to population coding, we repeated the above decoding analysis three times, each time removing neurons from one of the three functional classes, and compared decoding accuracies (permutation tests) to those obtained from a population with all classes, randomly downsampled to match the number of neurons. The magnitude of reduction in decoding accuracy due to such removal provided a measure of the contribution of each neuronal class to population coding. As expected, decoding of perceived directions was significantly reduced by removal of perceptual and perceptual–mnemonic—but not mnemonic—neurons, and decoding of perceived directions was significantly reduced by removal of mnemonic and perceptual–mnemonic—but not perceptual—neurons (Fig. 4c). Surprisingly, the detrimental effect on direction decoding accuracy was at least three to four times larger after removal of perceptual–mnemonic neurons than either perceptual or mnemonic neurons. This suggests that perceptual–mnemonic neurons—rather than perceptual or mnemonic neurons—contribute the most to population coding of perceived and memorized motion directions, and is consistent with our observation that the direction discriminability of perceptual–mnemonic neurons is higher than that of perceptual and mnemonic neurons (Fig. 3c). Lastly, removal of perceptual–mnemonic or mnemonic—but not perceptual—neurons caused a significant reduction in task decoding accuracy (Fig. 4c).

### Similarity between perceptual and mnemonic coding

An interesting pattern of decoding was observed for trials with incorrect task classification, represented by the upper right and lower left quadrants of the confusion matrix in Fig. 4a: squares along the descending diagonals of these quadrants showed a higher incidence of decoding than the remaining squares, indicating that when the decoder misclassified trials as belonging to the wrong task, it was still more likely to classify their sample direction correctly than incorrectly. We quantitatively confirmed this observation by measuring the decoder's performance at correctly classifying direction given incorrect task decoding. Direction decoding accuracy among trials with incorrect task classification was far above chance for both tasks (Fig. 4b; randomized trial labels tests, *P*<0.001 for both). This suggests a similarity between the LPFC population activity patterns that encode a direction when perceived and when memorized. However, if perceived and memorized directions were encoded by identical patterns, trials with correct and incorrect task decoding would be classified into similar direction conditions, and therefore would show similar direction decoding accuracy. Instead, we found that direction decoding accuracy in both tasks was significantly lower in task-error trials than task-correct trials (Fig. 4b; bootstraps; *P*<0.001), an indication of differences in the activity patterns coding for perceived and memorized directions.

If the population activity patterns coding for a perceived and a memorized direction are indeed similar, then a decoder trained to classify directions using the activity pattern from one task (perceptual or memory) should be able to correctly decode directions when tested in trials of the other task. To test this hypothesis, we performed linear discriminant analysis using training and testing sets that belonged either to the same task or to different tasks, using a 2-fold cross-validation method. This led to four measures of decoding accuracy based on all combinations of training/testing sets: perceptual/perceptual, memory/perceptual, memory/memory and perceptual/memory (Fig. 4d). As hypothesized, direction decoding accuracy was far above chance even when training and testing sets belonged to different tasks (randomized trial labels tests, *P*<0.001 for all four training/testing sets). These results strongly suggest that in LPFC, the population activity patterns coding for perceived motion directions resemble those coding for the same directions when memorized. This is likely due in part to the contribution of perceptual–mnemonic neurons with similar responses to perceived and memorized directions (Fig. 2e,f). However, the decoder's performance at decoding direction during a particular task was significantly higher when it was trained with trials of the same task than trials of the other task (Fig. 4d, bootstraps, *P*<0.001 for both tasks). This indicates that the population codes representing perceived and memorized directions partially differ. It is worth noting that while decoding performance was higher in the perceptual/perceptual (62.5%) set than the memory/memory set (60.5%), this difference only bordered significance (bootstrap, *P*=0.068), suggesting that the higher decoding accuracy for perceived than memorized directions reported in Fig. 4b likely represents a modest difference.

The differences between perceptual and mnemonic population codes could be due to neurons differing in either direction preference or overall firing rate levels between the two tasks. The distribution of direction preferences among all direction-selective neurons was similar between the two tasks; among neurons selective in both tasks, a majority showed no difference between their preferred directions in both tasks (Supplementary Fig. 4a,b). However, a fraction of neurons did show a difference; interestingly, this fraction was higher for differences of 90° than 180°, indicating that orthogonal shifts are more common than reversals in directionality between tasks. We then compared overall firing rates between the two tasks within each neuron. While no difference was found in a majority of neurons, this difference was significant in 36% of the neurons (Supplementary Fig. 4c,d; *t* tests corrected for multiple comparisons). The task-invariance of both direction preference and overall firing rates in a majority of neurons helps to explain the observed generalization in population decoding between perceived and memorized directions (Fig. 4d); on the other hand, differences in both direction preference and overall firing rate between tasks in a fraction of the neurons may account for the ability of the population to distinguish between perceived and memorized directions.

### Temporal dynamics of population coding

**Figure 5 f5:**
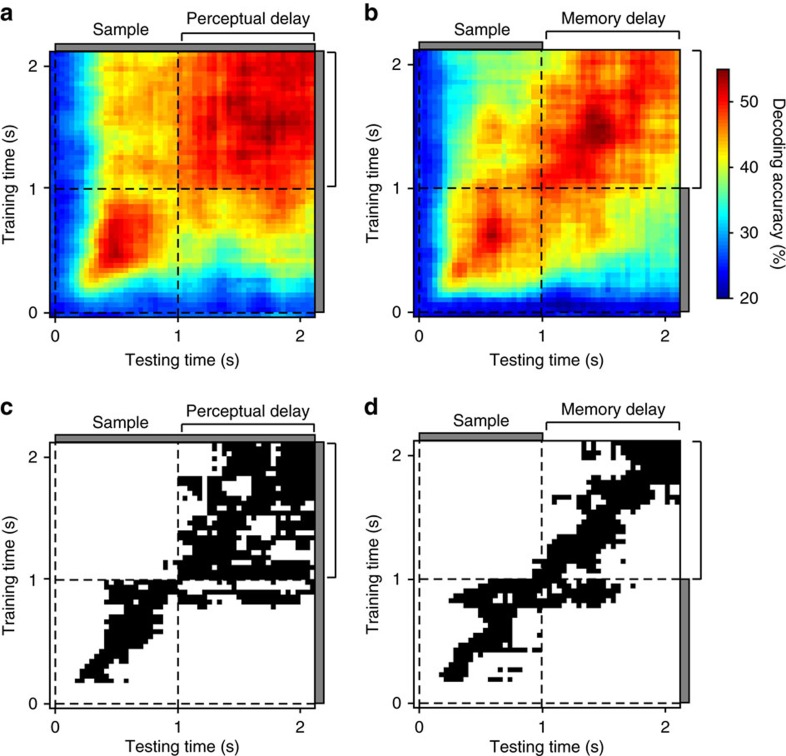
Cross-temporal population decoding of motion direction. (**a**,**b**) Sample direction decoding accuracy (colour-coded) for each pair of training/testing time windows in the perceptual task (**a**) and memory task (**b**). (**c**,**d**) For each training time window (vertical axis), black cells represent testing time windows (horizontal axis) for which cross-temporal decoding accuracy was both significantly higher than chance and not significantly different from decoding accuracy in the same-window training/testing.

Previous studies have shown that LPFC population coding in tasks requiring working memory is highly dynamic^10^,^11^. To examine whether the activity patterns encoding perceived and memorized directions were consistent over time during the delay, we trained the decoder with the activity pattern of each consecutive 40 ms time window during sample and delay periods of each task and tested it using the activity of the same and other windows. When training and testing within the same time window (Fig. 5a,b, diagonal cells), decoding accuracy in both tasks remained at a relatively stable level throughout the delay period, showing that the representations of perceived and memorized directions were present in the population activity at all times throughout the delay.

To test whether the population code underlying these representations was consistent or dynamic over time, we examined decoding accuracy when training and testing were done with different time windows. Remarkably, in both tasks, there was above-chance decoding in virtually all training/testing time window pairs beginning 240 ms after sample onset and continuing through the entire delay period (Fig. 5a,b, non-diagonal cells; see Methods). This shows the presence of cross-temporal generalization throughout the sample and delay periods. However, for many training/testing time window pairs, decoding performance was lower than in within-window training/testing, suggesting that in these periods, the population activity patterns likely undergo dynamic changes. At each training window, we statistically compared the decoding accuracy obtained by testing within the same window and those obtained using other testing windows (bootstraps; see Methods). The percentage of testing windows with a non-significant difference (Fig. 5c,d, black regions) provides a measure of the temporal stability of the population code. During the perceptual delay, this percentage was 70% (equivalent to 780 ms), and was more than twice the percentage obtained in the memory task (33%, equivalent to 370 ms). This indicates that the population activity patterns coding for memorized directions are more temporally dynamic and less stable than those coding for perceived directions. One possible explanation is that the constant visual presence of the sample stimulus during the perceptual delay provides stability to the underlying neuronal population activity representing the sample direction. In contrast, the absence of such constant sensory input during the memory delay may lead to a more dynamic code. How such dynamic code is read out as a stable representation is an important question for future studies.

We observed that decoding generalization in the perceptual task was mostly confined to the perceptual delay period and did not extend to earlier times of the sample presentation period. This may be due to the fact that during the first 1,000 ms of sample presentation, monkeys had the uncertainty of whether the sample would remain in view or had to be memorized, given that trials of the two tasks were randomly interleaved. In contrast, after 1,000 ms of sample presentation in the perceptual task, monkeys had full certainty that the stimulus would remain available for the rest of the trial.

### Feature proximity in population coding of motion directions

It is well known that in early visual cortical areas selective for visual features, such as motion direction or orientation, the activity of neurons varies as a function of feature space^12^. Hence, the closer two features are in feature space, the more similar their underlying population activity patterns will be. One important question is whether such feature-proximity structure is also present in the population activity patterns of higher-level areas such as LPFC. Feature proximity has been observed in the activity profiles of LPFC neurons for visual motion speeds^13^ and vibrotactile frequencies, not only during sensory input, but also during working memory maintenance^14^.

One way to investigate this question is to measure the incidence of trials with incorrect motion direction decoding. If the LPFC population activity patterns representing two motion directions are more similar the closer these directions are to each other, then a decoder trained on such activity patterns will be more likely to misclassify a direction as a nearby direction than as a distant direction. Alternatively, if there is no relationship between the proximity of two directions and the similarity in the activity patterns representing them, direction decoding errors should be equally distributed among all incorrectly classified directions.

**Figure 6 f6:**
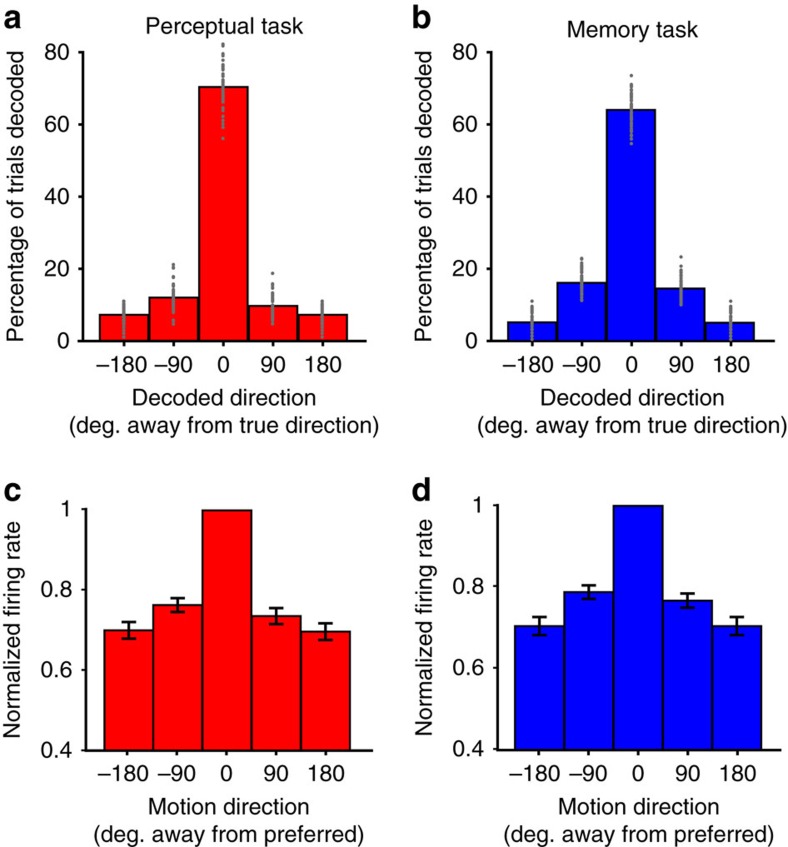
Parametric nature of LPFC motion direction representations. (**a**,**b**) Percentage of trials from the perceptual (**a**) and memory (**b**) tasks decoded as having a direction 0°, 90°, −90° or 180° away from their true direction. The value at 180° and −180° is identical and was duplicated for display purposes. Grey dots, performance from individual bootstraps. (**c**,**d**) Mean firing rate in the delay period of the perceptual (**c**) and memory (**d**) tasks across all directional neurons as a function of the sample motion direction with respect to each neuron's preferred direction, normalized to the preferred direction's firing rate. Grey dots, normalized firing rate of individual neurons; error bars, s.e.m.

As seen in Fig. 4a, for both tasks, direction decoding errors corresponding to directions adjacent to the true one (90° or −90° away) were more frequent than those corresponding to directions 180° away. In the perceptual task, the incidence of incorrect direction decoding was 56% higher for errors 90° away from the true direction than for errors 180° away (Fig. 6a; bootstrap, *P*<0.001); in the memory task, it was 194% higher (Fig. 6b; bootstrap, *P*<0.001). To confirm that this effect was specifically due to the relationships between the four directions along feature space, we destroyed such relationships by randomly swapping the direction labels between the four sample direction conditions within each task (while keeping trials grouped within condition), and repeated the decoding analysis. While this manipulation did not affect decoding accuracy for task or direction (Supplementary Fig. 5a), it did eliminate the significant difference between the incidences of direction decoding errors of 90° and 180° in both tasks (Supplementary Fig. 5b,c). Taken together, these results suggest that the activity patterns of the LPFC neuronal population show a feature-proximity structure whereby more proximal directions are represented by more similar patterns.

The above result suggested that, at least for a majority of neurons, each neuron's activity for its preferred direction should be more similar to the activity for directions 90° away than 180° away. Indeed, across neurons, firing rates were significantly lower in trials with a direction 180° than 90° away from the preferred, in both the perceptual task (Fig. 6c, paired *t* test, *t*(75)=2.02, *P*=0.023) and the memory task (Fig. 6d, paired *t* test, *t*(81)=2.13, *P*=0.018). Furthermore, across all neurons, firing rate differences between the preferred direction and the anti-preferred (180° away) were 2.9 times larger than the differences between the two other directions (90° and −90° away) in the perceptual task, and 2.3 times larger in the memory task. This indicates that for most neurons, the activity to both orthogonal directions was intermediate between the activity to the preferred and anti-preferred directions. While these results are consistent with the idea that LPFC neuron activity profiles may follow direction tuning curves, the four directions used in our experimental design are insufficient to fully assess the existence of direction tuning functions in LPFC neurons. However, a previous study using a similar task with more directions found LPFC neurons with responses suggestive of such tuning^15^.

### Relationship between neuronal activity and task performance

**Figure 7 f7:**
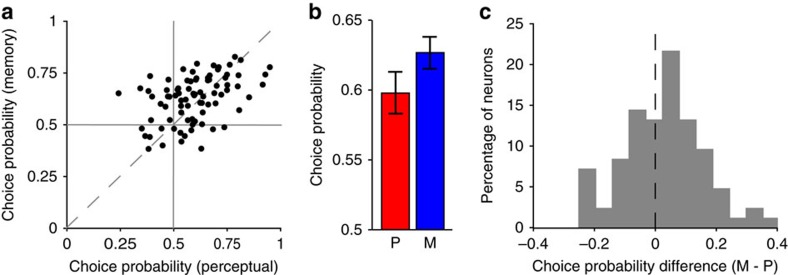
CP of delay activity during the perceptual and memory tasks. (**a**) CP of each directional neuron during the delay period of the perceptual and memory tasks (horizontal and vertical axes, respectively). Solid lines, chance CP; dashed line, unity line. (**b**) Mean CP (±s.e.) across neurons during the delay period of the perceptual task (red) and memory task (blue). (**c**) Histogram showing the distribution of within-neuron CP differences between the memory and perceptual tasks, among all directional neurons. Dashed line, no CP difference.

We next examined whether the delay period activity of LPFC neurons plays a role in performance of the perceptual and memory tasks. We applied choice probability (CP) analysis to quantify how well the behavioural outcome in each trial (correct or incorrect response) could be predicted from the activity of direction-discriminating LPFC neurons during the delay period. Across neurons, CP in both the perceptual task (mean CP=0.60) and the memory task (mean CP=0.63) was significantly higher than expected by chance (Fig. 7a,b; one-sample Wilcoxon signed-rank tests, *P*<0.001 for both tests). Interestingly, when comparing each neuron's CP between tasks, it was significantly higher in the memory than in the perceptual task across neurons (Fig. 7c; Wilcoxon signed-rank test, *P*=0.038). These results suggest that neuronal activity in LPFC is linked to task performance in both tasks, and more strongly during memory than during perception. Interestingly, this contrasts with our observation of stronger population coding of perceived than memorized directions in correct trials (Fig. 4b).

### Topography of neurons with perceptual and mnemonic coding

**Figure 8:**
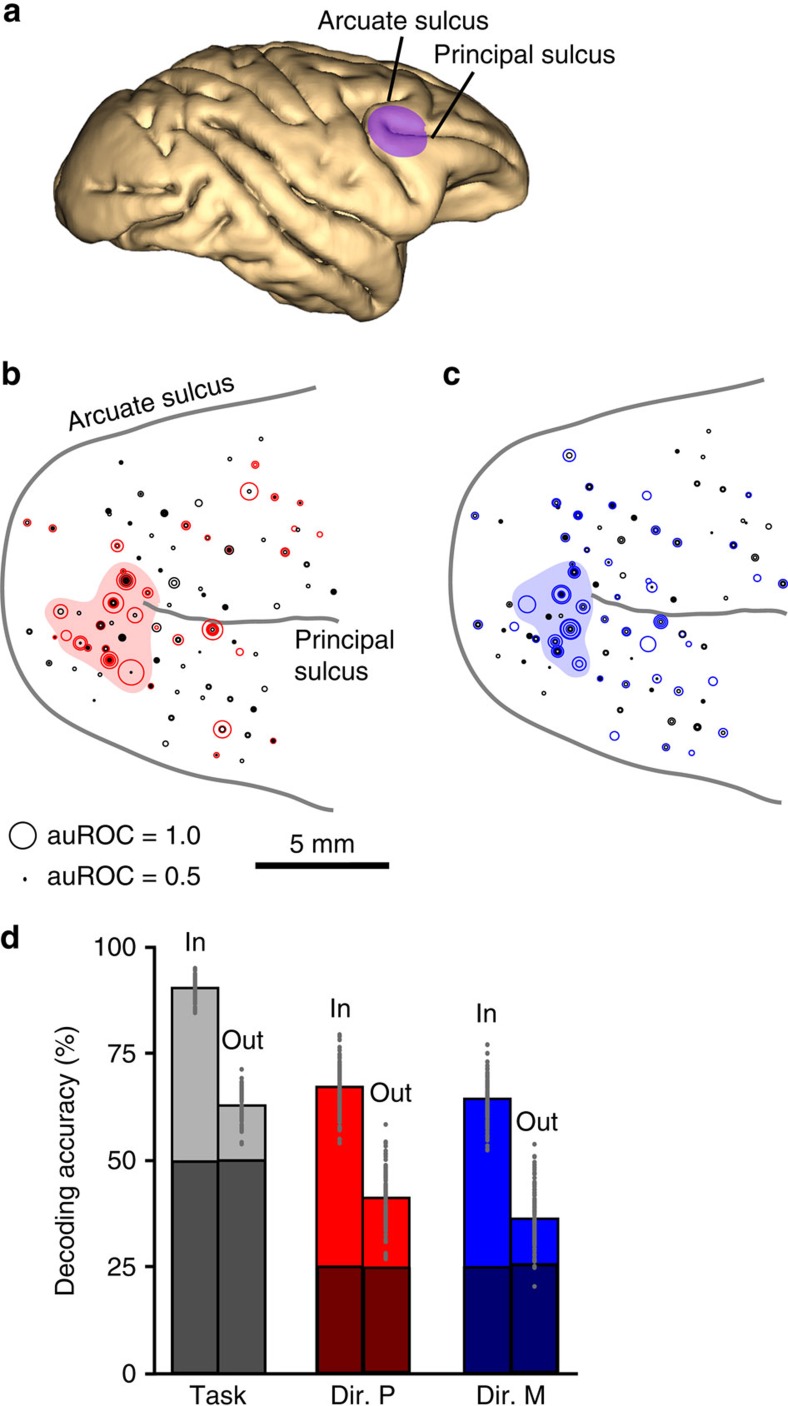
Anatomical location of LPFC neurons with perceptual and mnemonic coding. (**a**) MRI-based three-dimensional reconstruction of cortical surface showing LPFC (purple). (**b**,**c**) Location of all recorded neurons with respect to the arcuate and principal sulci. Each neuron is depicted as a ring, and its corresponding direction discriminability during the perceptual delay (**b**) and memory delay (**c**) is represented by the ring's diameter. Neurons with significant perceptual or mnemonic discriminability are labelled with red or blue, respectively. Multiple neurons were recorded from each location. Shaded areas show sub-regions with a cluster of high-discriminability neurons. Scale bar, 5 mm. (**d**) Percentage of trials decoded as the correct task (grey) or the correct sample direction during the perceptual task (red) or memory task (blue), using only neurons inside or outside the described sub-region. Conventions as in Fig. 4b.

Lastly, we examined the topographical organization of the recorded neurons across the prefrontal cortical surface in relation to their strength of perceptual and mnemonic coding, independently for each monkey. Interestingly, in both monkeys, the great majority of neurons with high perceptual discriminability (Fig. 8b, auROC>0.75), as well as those with high mnemonic discriminability (Fig. 8c), were clustered within a sub-region around the posterior end of the principal sulcus (red shaded area): the mean distance between pairs of neurons was significantly lower than expected by chance for neurons with either high perceptual (permutation test; Monkey Mi: *P*=0.04; Monkey Se: *P*=0.03) or mnemonic (permutation test; Monkey Mi: *P*=0.021; Monkey Se: *P*<0.001) discriminability (see Methods). Interestingly, compared to the results obtained with the entire population (Fig. 4b), decoding accuracies for task, perceived directions or memorized directions were similar when only using neurons inside the sub-region, but substantially reduced when only using neurons outside (Fig. 8d). This difference was present despite the number of ‘outside' neurons being twice that of ‘inside' neurons, and remained after randomly downsizing the number of ‘outside' neurons to equal the number of ‘inside' neurons. This shows that most of the contribution to the population code comes from neurons concentrated inside the observed sub-region.

These results showed that coding of perceived and memorized directions is not evenly spread across all neurons in the explored area but mainly concentrated within a relatively small sub-region. The position and extent of this strongly coding sub-region was similar for perceptual and mnemonic coding, even after removing all perceptual–mnemonic neurons. This suggests that there is no anatomical segregation between perceptual and mnemonic coding neurons at the millimetric scale studied here, at least. Whether such segregation occurs at the scale of smaller cortical columns or across layers within columns remains to be examined.

## Discussion

In the present study, we investigated how coding of perceived and memorized features are distributed across the population of LPFC neurons. Neurons showed a wide variety of combinations of coding strength for perceived and memorized directions, with some neurons preferentially or exclusively encoding one of these two types of representations and others encoding both. We further showed that when combining such diversity of responses, the resulting population activity pattern contained enough information for a linear discriminant classifier to effectively decode both perceived and memorized motion directions, and to determine the nature of the representations—perceived or memorized. Although neurons with direction-selective properties were found across a relatively large area of LPFC, neurons with the strongest perceptual and mnemonic feature coding were clustered within a sub-region around the posterior end of the principal sulcus corresponding to the cytoarchitechtonic area described as 8 Av (ref. 8). Interestingly, this sub-region has been specifically shown to send direct feedback projections to motion direction-selective areas middle temporal (MT) and medial superior temporal (MST)^16^, and has been proposed as a likely source for feature attention^17^.

Previous studies have reported that cortical ablations^18^ and pharmacological manipulations^19^,^20^,^21^ in LPFC impair monkeys' performance of oculomotor delayed response tasks when the cue stimulus becomes perceptually unavailable and must be memorized, but not when the same stimulus remains present. These results have suggested that LPFC plays a more important role in tasks that rely on working memory representations than those relying only on perceptual information. These studies seem at odds with others reporting that more LPFC neurons encode perceived than memorized spatial locations^22^,^23^,^24^. In our study, we found that while perceptual and mnemonic representations of a non-spatial visual feature were preferentially encoded by similar proportions of neurons, population decoding of perceived features was higher than memorized features. Interestingly, however, the activity of LPFC neurons was more strongly linked to performance of the memory task than the perceptual task. Therefore, our results do not suggest that LPFC function is preferentially associated with perceptual or mnemonic coding overall.

Functional imaging studies in humans have revealed that overlapping brain regions, including lateral prefrontal regions, are activated while subjects perceive and attend to visual stimuli, and while they maintain visual representations in working memory^25^,^26^. These results can be explained by our observation that neurons with the strongest perceptual and mnemonic coding are present within a small region of LPFC (Fig. 8b,c), and that a fraction of the neurons (about 1/3) shows both types of coding. The limited spatial resolution of functional imaging may not allow discerning between the intermingled populations of neurons that preferentially or exclusively encode one of these two types of representations.

Current models propose that persistent working memory-related activity is supported by recurrent excitatory connections between neurons—a cortical architecture characteristic of high-order association areas such as LPFC^27^,^28^,^29^. On the basis of our results, it is possible that sensory inputs are fed into such a network via perceptual and perceptual–mnemonic neurons, and on stimulus disappearance, persist through a recurrent excitatory network of mnemonic and/or perceptual–mnemonic neurons. Furthermore, it is possible that the resilience of working memory representations in LPFC to distractor interference^30^ is mediated by exclusively mnemonic neurons that do not respond to visual inputs (Fig. 2c,d). Importantly, our results do not support models in which persistent activity is stable over time. In most neurons, feature-selective delay activity showed temporal dynamics over time that were consistent across trials. As a result, the population activity pattern at each time window could be used to reliably decode memorized directions, but decoding did not generalize across time windows. Therefore, in the context of working memory, the term sustained activity should be understood not as ‘sustained at a stable level over time', but as ‘sustained at dynamic levels different from baseline firing'. Furthermore, the presence of neurons with exclusive coding during the memory but not the perceptual task challenges the notion that the activity of LPFC neurons during working memory results from the persistence of their response to the to-be-remembered stimulus after it becomes perceptually unavailable^31^. Our results thus suggest the need to re-conceptualize or expand current models of how the LPFC microcircuitry implements working memory encoding and maintenance.

It has been proposed that LPFC plays a fundamental role in the control of feature-based attention^32^. A working memory representation of a relevant feature, maintained in LPFC, is thought to serve as a top-down signal that modulates sensory processing in visual cortical areas^7^,^17^,^33^, ultimately prioritizing the perception of stimuli matching this template^34^,^35^,^36^. Whether such top-down modulatory mechanisms are carried out specifically by the memory-coding neurons observed in our study remains to be investigated. In turn, neurons with perceptual coding may play a role in maintaining currently perceived visual features within the focus of attention. Whether the perceptual neurons characterized here specifically encode attended features or represent all visual inputs regardless of attention cannot be fully determined by our study, given that our task was not designed to require animals to maintain attention on the sample direction during the perceptual delay.

Numerous studies using retro-cues in humans have demonstrated that attention can serve to prioritize not only a behaviourally relevant stimulus among multiple concurrent stimuli, but also a relevant working memory representation among multiple simultaneously maintained representations^37^,^38^,^39^,^40^. Yet the underlying mechanisms at the level of neuronal ensembles remain poorly understood. One important issue to address in future studies is the role of perceptual, mnemonic and perceptual–mnemonic neurons in tasks in which one representation is prioritized and monitored among multiple perceptual or mnemonic representations. Previous studies have shown that monkeys with lesions of LPFC have deficits in tasks that require monitoring multiple representations^41^,^42^. One possibility is that the neurons with perceptual and mnemonic coding characterized here serve to ‘read out' perceptual and mnemonic representations encoded in other brain areas^6^,^7^,^43^,^44^, keeping them within the focus of attention while avoiding distractor interference.

In our study, the generalization of decoding for motion direction between the perceptual and memory tasks suggests that population activity patterns encoding perceived and memorized visual features are similar to a degree. However, such generalization was not complete, indicating that the population codes representing perceived and memorized directions differ to a certain extent. This was accounted for by differences in both direction preference and overall firing rates between tasks in a fraction of the neurons, and may represent a mechanism for LPFC to distinguish features of currently perceived visual stimuli from those held in working memory. It is possible that this mechanism may allow the brain to represent perceptually available stimulus features while concurrently maintaining representations in working memory without confounding them. This ability may depend on a fine balance in the activity patterns of perceptual, mnemonic and perceptual–mnemonic neurons. Loss of this balance (for example, abnormal activation of perceptual neurons in the absence of visual stimulation) may be a source of hallucinatory experiences typical of mental disorders such as schizophrenia. Some studies have proposed a somewhat similar mechanism for the origin of ‘hallucinatory representations' in neural network models^45^. Interestingly, functional imaging studies in humans have found abnormal activation in the LPFC of patients with schizophrenia^46^, and at least one investigation reported cessation of hallucinations in schizophrenic patients as a consequence of removing part of LPFC and/or its connections^47^. Future studies manipulating the activity of LPFC neurons will be required to further examine this.

In summary, the activity patterns of the LPFC neuronal population representing perceived and memorized visual features may serve as a substrate for the brain to monitor these two types of representations and to discriminate between them. It may also serve to integrate perceptual and mnemonic information, particularly during tasks that require comparisons between current and past sensory experiences.

## Methods

### Animals

Two adult male rhesus monkeys (*Macaca mulatta*), 10 and 11 years old and weighing 8 and 9 kg, participated in the experiments. Before the experiment, monkey Mi had not received any behaviourally training, and monkey Se had been trained in an unrelated task. Experimental sessions were carried out between 1,300hours and 1,900 hours. The monkeys were given fruit juice as a reward for correctly performing each task trial, totalling a daily intake of between 300 and 600 ml. At the end of each training and recording session, they were also given a portion of fruits in addition to their daily food ration. We measured their body weights daily to ensure stable health tasks. All animal procedures complied with the Canadian Council of Animal Care guidelines and were approved by the McGill University Animal Care Committee.

### Visual Stimuli

Visual stimuli were generated using a custom-made software on an Apple G4 Power computer, and were back-projected onto a screen using a NEC WT610 video projector (1,024 × 760 pixels resolution, 85 Hz refresh rate). The monkeys were positioned 57 cm away from the screen. Sample and test stimuli were composed of random dots moving linearly with 100% coherence and within a virtual circular aperture (13 cd m^−2^, dot luminance contrast, 0.17° dot size, density of 4 dots per squared degree). The dots' speeds varied between 2 and 32° s^−1^ across sessions (8 or 16° s^−1^ in most sessions). The motion directions of the sample and tests were chosen from a set of four directions separated by 90° (where one of the directions in the set was either 0°, 30° or 60°). It is important to clarify that we recorded from neurons in area MT simultaneously with those in LPFC (for purposes outside of the scope of the current study); motion speed and directions were chosen to match the feature preference of MT neurons but were random with respect to the LPFC neurons' feature preferences. This conveyed the advantage of avoiding a biased overestimation of direction discriminability in the population of LPFC neurons. There was no relationship between the functional classification of neurons and the speed chosen for stimuli on each session. The set of motion directions was constant throughout each recording session and identical for both tasks, but was changed from session to session and often within the same day. Thus, the monkeys could neither use long-term memory representations of these directions nor simply learn four fixed categories across sessions as a strategy to solve the task.

### Behavioural task

Monkeys were trained to perform two versions of a match-to-sample task (Fig. 1a). During all trials, the monkeys maintained their gaze on a white fixation square (size 0.25° × 0.25°) positioned at the centre of the screen and pressed a button to initiate a trial. After 470 ms of successful fixation, a sample stimulus moving in one of four possible directions was presented.

### Memory task

For trials of the memory task, the sample was removed 1,000 ms after its presentation, and the monkey was required to memorize its motion direction. After a delay period of randomly varied duration between 1,200 and 2,000 ms, a test stimulus was presented for 590 ms. In half of the trials, the motion direction of the test matched that of the sample, and the monkey was required to recognize this match and release the button to receive a juice reward. If the monkey failed to do so, the trial was terminated without a reward. In the remaining half of the trials, the test direction did not match the sample; the monkey had to continue to hold the button and release it when a second test stimulus matching the sample direction was presented. A behaviourally irrelevant stimulus with 0% coherent motion and lower luminance contrast was presented simultaneously with the test stimuli on the opposite hemifield. The locations of the test and irrelevant stimuli, both different from the sample location, were randomly swapped from trial to trial. Therefore, the monkey could not know the test location before its presentation.

### Perceptual task

Trials from the perceptual task were identical to those from the memory task in every aspect, except that the sample remained on the display until the end of the trial. This allowed the monkeys to have visual access to the sample direction even during the presentation of the tests; therefore, they were not required to memorize the sample direction.

### Eye positions

During all sessions, eye position signals were sampled at a frequency of 200 Hz using a video-based eye tracker system (Eye Link 1000, SR Research, Kanata, ON, Canada). Monkeys were allowed to start a trial only when their gaze position fell within a 1° radius around the fixation point centre. The trial was terminated without a reward if their gaze position moved outside this radius at any time before the end of the trial.

### Surgical preparation of the monkeys

Monkeys were implanted with titanium head posts to stabilize their heads during recordings. Each monkey was also implanted with a circular Cilux recording chamber 20 mm in diameter (Crist Instruments, MD, USA). The chamber was positioned on top of a circular craniotomy of the frontal bone, of similar diameter to the chamber, that provided access to the LPFC in the right hemisphere—specifically, the region anterior to the arcuate sulcus and around the posterior end of the principal sulcus (Fig. 8). The stereotactic coordinates of the craniotomy centre were 30 mm anterior and 17 mm lateral.

### Anatomical localization of recording sites

A magnetic resonance imaging (MRI) scan was conducted on each monkey before the surgery to guide the positioning of the chamber. After chamber implantation and during recordings, a plastic grid (Crist Instruments, MD, USA) was positioned on top of the recording chamber. Five glass capillaries filled with mineral oil were positioned parallel to electrode trajectories at four different grid locations, one at the centre and one in each cardinal location that served as a reference for electrode trajectories. With this preparation in place, an additional MRI was conducted to precisely locate the positions of the brain areas of interest with respect to the electrode trajectories. The boundaries of LPFC were then identified in the monkeys' MRIs. LPFC neurons were recorded by placing the electrode tip in positions around the principal sulcus, anterior to the arcuate sulcus (Fig. 8b,c). The location of each recording site, along the cortical surface, was determined using the coordinates of the electrode position in the grid with respect to the coordinates of the reference electrode trajectories (oil-filled capillaries) in the MRI. It should be noted that virtually all 272 neurons were recorded within a region that is clearly outside the frontal eye field based on our MRI mapping method, with the exception of approximately five neurons, which could be at the border between areas.

To quantitatively test whether neurons with high sensory discriminability were closer together than would be expected from a random anatomical distribution across the recorded cortical surface, we performed the following permutation test independently in each monkey: we measured the distance between all pairs of neurons that possessed high sensory discriminability (auROC>0.75) and calculated the mean among all of these distances. We then computed ‘surrogate' distances between the same neurons after randomly shuffling the position values among all recorded neurons, and repeated this procedure 1,000 times while computing a surrogate mean distance between pairs of neurons for each repetition. The observed mean distance was then compared to the 1,000 mean surrogate distances, and the probability (*P* value) of obtaining the observed mean distance, given a random distribution of the neurons, was then computed as the percentage of surrogate values lower than the observed value. The same procedure was repeated for neurons with high mnemonic discriminability.

### Electrophysiological recordings

During each experimental session, transdural penetrations were made with standard epoxy-insulated extracellular tungsten electrodes (FHC Inc, Bowdoin, ME, USA; shank diameter=500 μm; impedance=2–4 MΩ at 1 kHz). A blunt guide tube, positioned 5–10 mm from the recording electrode(s) and touched but did not penetrate the dura, served as the reference. During each session, we simultaneously recorded with one to four electrodes separated by at least 2 mm. We used a Plexon data acquisition system (MAP) to simultaneously record and store spike and LFP data (Plexon, Dallas, TX, USA). The electrode signal was passed through a head stage, with unit gain, and then split into the spike and the LFP components. For spike recordings, the signal was filtered between 250 and 8,000 Hz, amplified, and digitized at 40 kHz. Single neuron spiking activity was then isolated using Plexon online and offline sorting software. Our study only included units that were clearly identified as single units—those whose spike form properties had clear clustering and isolation from those of other units. Multiunit activity was excluded.

### Data analysis

Data analysis was performed using custom software written in Matlab (MathWorks). All analyses were conducted on data recorded in correctly performed trials, unless otherwise indicated. Results obtained from both monkeys were qualitatively similar. For each trial, we computed each neuron's mean firing rate during the perceptual delay or memory delay period. All analyses excluded the first 240 ms of the delay period to avoid confounding residuals of the sensory response to the sample on working memory-related activity during the memory task. Because the duration of the perceptual and memory delays could vary between 1,200 and 2,000 ms across trials, we only analysed activity during the first 1,200 ms of both delays. Trials were grouped by task and by sample direction. All statistical tests were chosen based on standard requirements. When data did not follow the appropriate assumptions of parametric tests, non-parametric tests or permutation tests were used.

### Receiver operating characteristic analysis

For each neuron, we performed receiver operating characteristic (ROC) analyses to quantify its ability to discriminate among sample motion directions (direction discriminability). The following analyses were performed independently on the perceptual and memory tasks, and during the perceptual delay and memory delay periods, respectively. We computed the area under the ROC curve (auROC) to measure the separability of the distributions of mean firing rates to each motion direction in all trials, between all possible pairs of sample directions; the highest value was used as a measure of the neuron's direction discriminability, and the direction with the highest firing rate was chosen as the preferred direction, independently in each task. to compare auROC values between all pairs of directions within each neuron, as well as between tasks within and across neurons, auROC values between 0 and 0.5 were rectified to their corresponding values in the range between 0.5 and 1 (for example, a value of 0.1 was rectified to 0.9).

To test whether discriminability (auROC) was significantly higher than expected by chance, we performed a permutation test in which the above procedure was repeated completely after randomly shuffling the direction labels of all trials. This was repeated 500 times to yield 500 surrogate auROC values. If the real auROC reached or exceeded the 99th percentile of the distribution of the 500 shuffled surrogates, the auROC was considered significant. This significance threshold was adjusted for multiple comparisons resulting from testing significance across all neurons. We used Wilcoxon rank sum tests to compare between two distributions of auROC values across neurons for the same task. To test whether the percentage of perceptual–mnemonic neurons was significantly higher than expected if the probabilities of each neuron showing perceptual and mnemonic discriminability were independent of each other, we performed the following permutation test: among perceptual, perceptual–mnemonic and mnemonic neurons, we randomly reshuffled the labels of significant perceptual and mnemonic discriminability between them, and measured the percentage of neurons that were simultaneously labelled with significant perceptual and mnemonic discriminability (surrogate value). We repeated this shuffling procedure to obtain a total of 10,000 surrogate values for the percentage of perceptual–mnemonic neurons. The real percentage obtained from our data was then compared to the 10,000 surrogates, and the *P* value of the test was obtained from its rank among the surrogates.

We computed the Pearson's correlation coefficient between perceptual and mnemonic discriminability across neurons, where the *P* value represented the probability of obtaining a correlation as large as the observed value by chance if the true correlation is zero. Chance levels of correlation were obtained after shuffling perceptual and mnemonic discriminability values between neurons.

The distributions of perceptual and mnemonic discriminability across neurons were similar in the two monkeys (Supplementary Fig. 1). All other results derived from discriminability values, as well as all decoding results, were consistent between the two animals. We therefore present results obtained by combining all neurons from both monkeys.

We also computed for each neuron the proportion of explained variance (*η*^2^) obtained from one-way ANOVAs comparing the delay period responses in the four sample direction conditions of each task, where *η*^2^ is the between-conditions sum of squares divided by the total sum of squares (Supplementary Fig. 2).

### Choice probability

For each neuron possessing delay-period direction selectivity, we obtained the mean firing rate during the delay period of all correct and error trials when the sample moved in the neuron's preferred direction. We selected these trials based on previous observations that the highest CP for each neuron results when the relevant motion direction is the neuron's preferred direction. Using these values, we computed CP, that is, the auROC between the firing rates of correct and error trials. This was done independently for the perceptual and memory tasks. One-sample Wilcoxon signed-rank tests (one-tailed) were first used to test whether mean CP across neurons was significantly higher than 0.5. Then, to test whether CP was significantly higher in the memory than the perceptual task within neurons, we compared the difference between CP in the two tasks (memory CP−perceptual CP) against 0 using a one-tailed Wilcoxon signed-rank test. Neurons with less than five correct or five error trials (preferred-sample) were excluded from the analysis.

### Population decoding analyses

Linear discriminant analysis was performed to quantify the ability of the population of recorded neurons to encode, during the delay period, the nature of the sample representation—whether it was being perceived or memorized (that is, the task condition), as well as its feature value—which of the four motion directions was being represented. The analysis was performed using the Matlab function ‘classify' and the diagLinear option. For each of all 272 neurons recorded, we randomly selected 30 trials per sample direction condition in each of the two tasks. This resulted in a total of 240 trials for all eight conditions together. To create the pseudopopulation, trial simultaneity among neurons was randomly assigned between trials of the same condition. This analysis required neurons with at least 30 trials recorded from each and all of the eight conditions. Overall, 105 neurons met this criterion. We applied linear discriminant analysis to decode the task and sample direction condition of each trial from the average firing rates of all the neurons during the delay period of each task, employing a leave-one-out cross-validation method for training and testing. For example, decoding applied to all eight conditions used 239 trials for training the discriminant and one for testing, where each time a different trial of the 240 was used as a test trial. Virtually identical results were obtained when repeating our analyses employing a method with balanced numbers of trials from each condition for training and testing (that is, 25 training trials and 5 testing trials per condition).

Before training, we *z*-scored all firing rates for each neuron and performed feature pre-processing on the training set of trials; the pre-processing consisted of a one-way ANOVA with eight levels corresponding to the eight conditions, to select neurons with a significant main effect of condition on firing rates^48^. Neurons that did not fulfil this criterion were excluded from training and testing. The number of neurons in the resulting pseudopopulation varied across runs due to the bootstrapping procedure described below, ranging between 35 and 43 neurons. Of these neurons, the percentage belonging to monkeys Mi and Se was on average 52% and 48%, respectively. Similar results were obtained using pseudopopulations composed of neurons recorded from each individual monkey (Supplementary Fig. 3).

The purpose of decoding analysis was to obtain an overall measure of how well LPFC neurons, pooled into a pseudopopulation of non-simultaneously recorded neurons, could discriminate between four motion directions separated by 90° (independently of which directions were chosen). Given such purpose, we aligned (that is, rotated) the direction reference frame labels across sessions so that one of the directions would be labelled as 0° and all others at 90° with respect to each other. For example, if in a given session we used directions of 30°, 120°, 210° and 300°, these directions were relabelled as 0°, 90°, 180° and 270° for both tasks. This allowed us to pool all sessions for decoding analysis.

For both tasks and each of the four sample direction conditions, we determined the number of trials that were decoded as belonging to a given task and sample direction condition (incidence of decoding). This resulted in an 8-by-8 confusion matrix (Fig. 4a). Decoding accuracy was then computed as the number of correctly decoded trials divided by the total number of correctly and incorrectly decoded trials. Trials were considered as having correct task decoding if they were classified as a condition belonging to the same task as the trial in question, regardless of the sample direction condition. Sample direction decoding was computed separately within trials with correct and incorrect task decoding.

We used a bootstrap procedure to estimate the variability of each decoding accuracy measure. From our original recorded neuronal population, we sampled with replacement to generate 100 bootstrap samples with the same number of neurons as the original population. Because each neuron's trials were shuffled within each condition before assigning trial simultaneity, there was no redundancy between duplicate neurons in the same trials. Decoding analysis was then performed on each bootstrap sample, generating 100 bootstrap values of decoding accuracy. To statistically compare a pair of mean decoding accuracy values, we ran an additional permutation test in which we computed the difference between the two decoding accuracies (real value) and compared it to 1,000 surrogate difference values, each computed after shuffling all decoding accuracy bootstraps between them. The proportion of surrogate values exceeded by the real value provided an estimate of the *P* value.

To statistically test whether a given mean decoding accuracy was significantly higher than expected by chance, we repeated the decoding procedure 1,000 times after shuffling the condition labels across trials each time to obtain 1,000 surrogate values of decoding accuracy. The proportion of surrogate values exceeded by the real-mean decoding accuracy provided an estimate of the *P* value (randomized trial labels test).

For cross-temporal decoding (Fig. 5), we performed the following analyses separately and identically for each of the two tasks, following similar steps as those described above. We trained the classifier to decode motion direction from the average firing rates of neurons in a given 40-ms time window, and then tested using the average firing rates of neurons in the same or a different time window, using a leave-one-out cross-validation method. For each pair of training/testing time windows during the sample and delay periods, we obtained direction decoding accuracy (Fig. 5a,b) and tested significant difference from chance accuracy using the permutation test described previously. For each training window, we used the bootstrap statistical analysis previously described to compare the decoding accuracy obtained by testing within the same window with those obtained using other testing windows (Fig. 5c,d). For all comparisons, statistical significance of the *P* value was set to 0.01, accounting for multiple time window comparisons according to control analyses performed over shuffled data.

The above decoding analysis was also repeated three times on the same population of neurons after removing neurons from each of the three functional classes (perceptual, perceptual–mnemonic and mnemonic). For each of the three populations above, we generated a sample size-matched population by randomly sampling neurons from the original population without replacement, and repeated the decoding analyses. Decoding accuracies for each of the three populations above were compared to those from their equivalent downsampled populations by subtracting the former from the latter, and performing the bootstrap tests described earlier to asses a statistical difference (Fig. 4c). Decoding accuracies were also computed for sub-populations that excluded neurons inside or outside the sub-region described in Fig. 8, and the above procedure was used to down-sample the number of ‘outside' neurons to equal the number of ‘inside' neurons.

Linear discriminant analysis was also performed on training and testing sets of trials belonging to the same task or to different tasks. For each task, we divided all trials into two sets of equal size. Testing was performed using a 2-fold cross-validation method in which the 120 total trials of the four direction conditions of each task were divided into 60 training trials and 60 testing trials. Statistical comparisons were performed as in the aforementioned analyses.

For each task, we independently measured the incidence with which trials were decoded as belonging to a condition with a sample direction that was the same as – or 90°, 180° or −90° away from—the true direction. The incidence values were first obtained separately for each sample direction condition before being averaged. To test whether the incidence of decoding was significantly higher for decoding errors of ±90° than for 180° away from the true direction, we averaged the incidence between 90° and −90° errors, and compared it to the incidence of 180° errors within each repetition of the decoding analysis and across the 50 repetitions using a paired *t* test. For the control analysis in Supplementary Fig. 5, we repeated the above procedure, this time randomly shuffling the direction labels of the four sample direction conditions for each of the 100 bootstraps (while keeping trials grouped within condition) before performing the decoding analysis.

Across all direction-selective neurons in each task, we used a paired-sample *t* test to compare, within neurons, the delay period firing rates between trials with directions 90° versus 180° away from each neuron's preferred direction (Fig. 6c,d). We also computed, for each neuron, the difference in mean firing rate between preferred and anti-preferred (180°) direction trials, and between 90° and −90° trials, and obtained the average ratio of the former to the latter across neurons.

We repeated all decoding analyses using a support vector machine classifier instead of linear discriminant analysis and obtained similar results. It is important to note that, because neurons were not recorded at the same time, the resulting estimates of decoding accuracy are approximations of a real, simultaneously activated neuron population's coding ability. However, it has been shown that the decoding accuracy obtained from a population of simultaneously active neurons is similar to, and in certain circumstances lower than, that obtained from a population of non-simultaneously active neurons^49^.

### Data availability

Main data and analyses code are publicly available on Open Science Framework, https://osf.io/ebm49/.

## Additional information

**How to cite this article:** Mendoza-Halliday, D. *et al*. Neuronal population coding of perceived and memorized visual features in the lateral prefrontal cortex. *Nat. Commun.* **8**, 15471 doi: 10.1038/ncomms15471 (2017).

**Publisher's note:** Springer Nature remains neutral with regard to jurisdictional claims in published maps and institutional affiliations.

## Supplementary Material

Supplementary Information Supplementary Figures
