# Supplementary material for: Neuronal population coding of perceived and memorized visual features in the lateral prefrontal cortex
Source: Nat Commun. 2017 Jun 1;8:15471. doi: 10.1038/ncomms15471 (PMC5461493; doi:10.1038/ncomms15471)
Supplement: Supplementary Information — Supplementary Figures [file ncomms15471-s1.pdf]

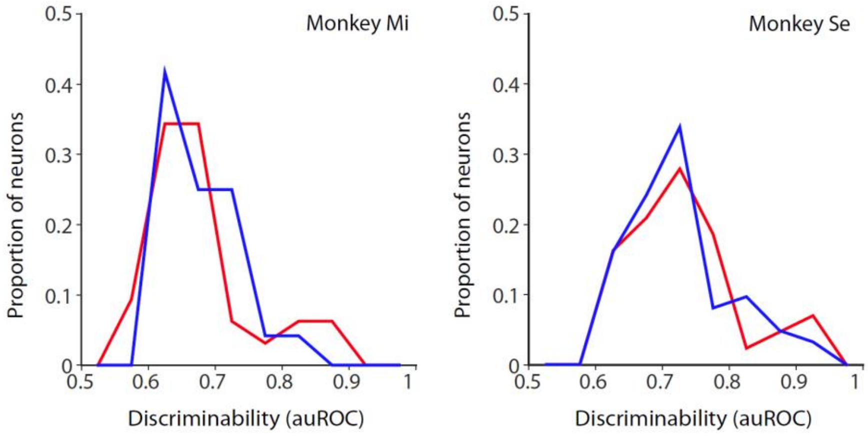


**Supplementary Figure 1.** Distribution of delay period discriminability (auROC) values across all direction-selective neurons recorded from each monkey in the perceptual (red) and memory (blue) tasks.

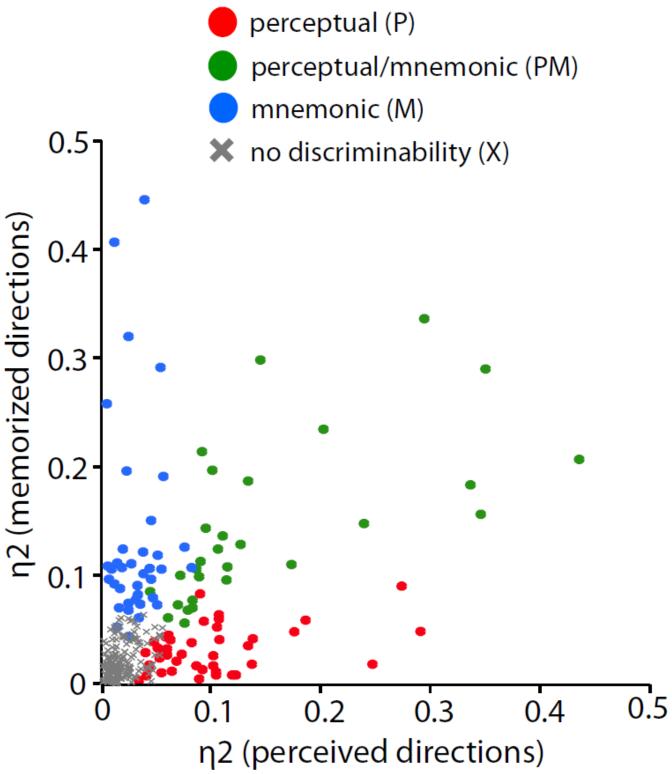


**Supplementary Figure 2.** Proportion of explained variance ($\eta^2$) of the responses between sample direction conditions during the perceptual (horizontal) and memory (vertical) delay periods, for all neurons (dots). Neurons were classified based on the significance of one-way ANOVAs across direction conditions in each task (color label).

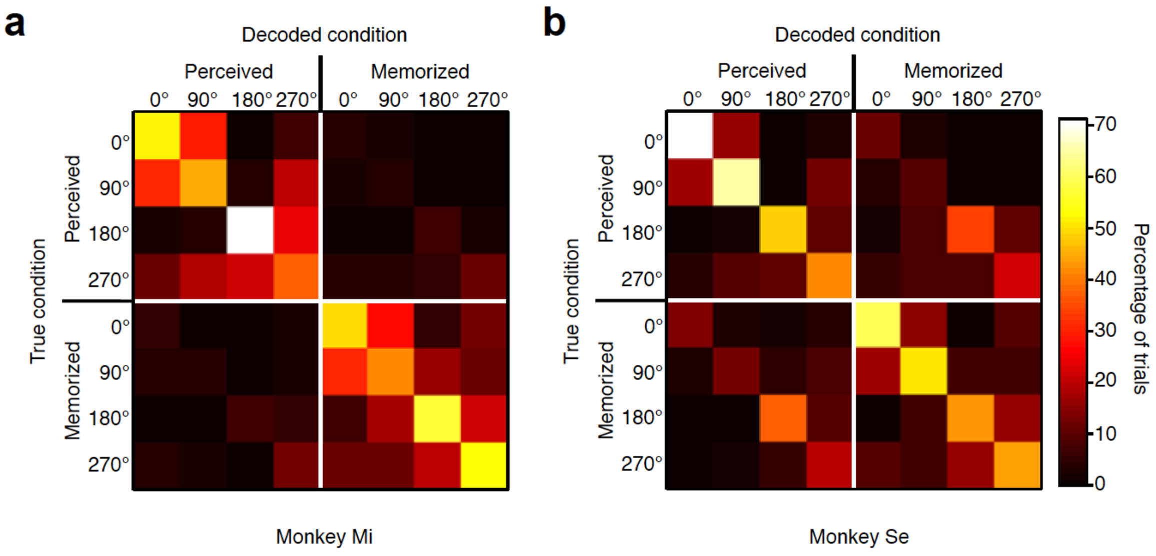


**Supplementary Figure 3.** Population decoding of task and motion direction for each individual monkey. Confusion matrix showing the percentage of trials of a given condition (true condition) that were decoded as belonging to each of the eight conditions (decoded condition), obtained from neurons recorded in monkeys Mi (a) and Se (b).

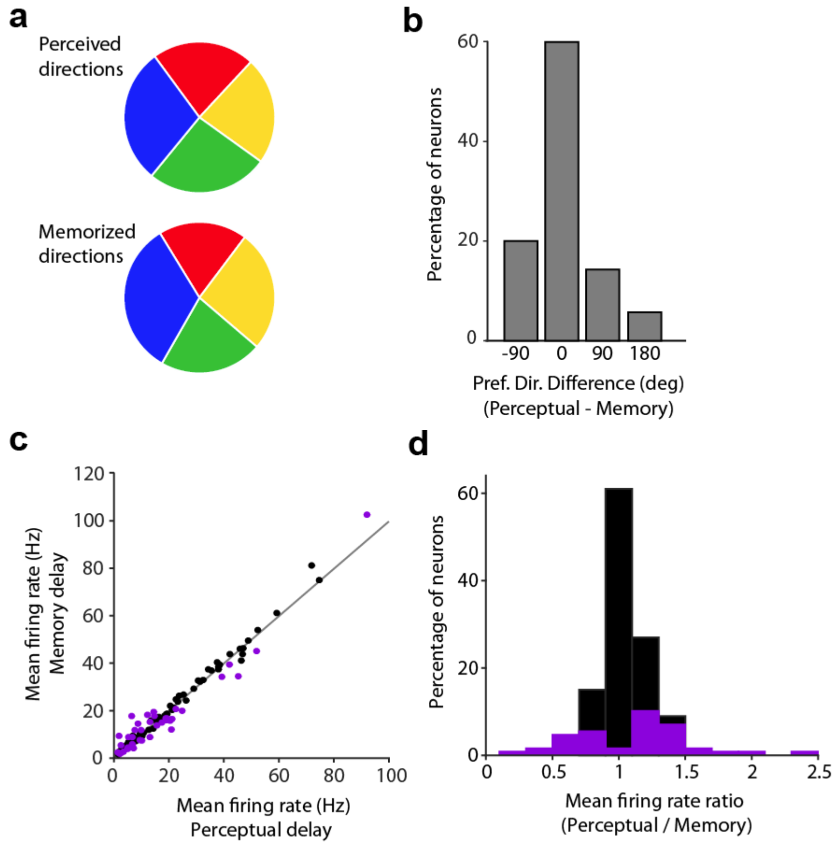


**Supplementary Figure 4. Comparison of direction preference and mean firing rate between the perceptual and memory tasks.** (a) Pie charts showing distribution of preferred directions (color-coded as in **Fig. 1**) among neurons with selectivity for perceived (top) and memorized (bottom) directions. (b) Distribution of preferred direction difference between tasks (perceptual – memory) in degrees, among direction-selective neurons. (c) Scatter plot of the mean firing rates in the delay period of the perceptual task (horizontal axis) and memory task (vertical axis) for all neurons (dots). (d) Frequency histogram showing distribution of mean firing rate ratios between tasks (perceptual / memory) among all neurons. Bars are vertically stacked. In (c) and (d), neurons with significant difference between tasks are shown in purple.

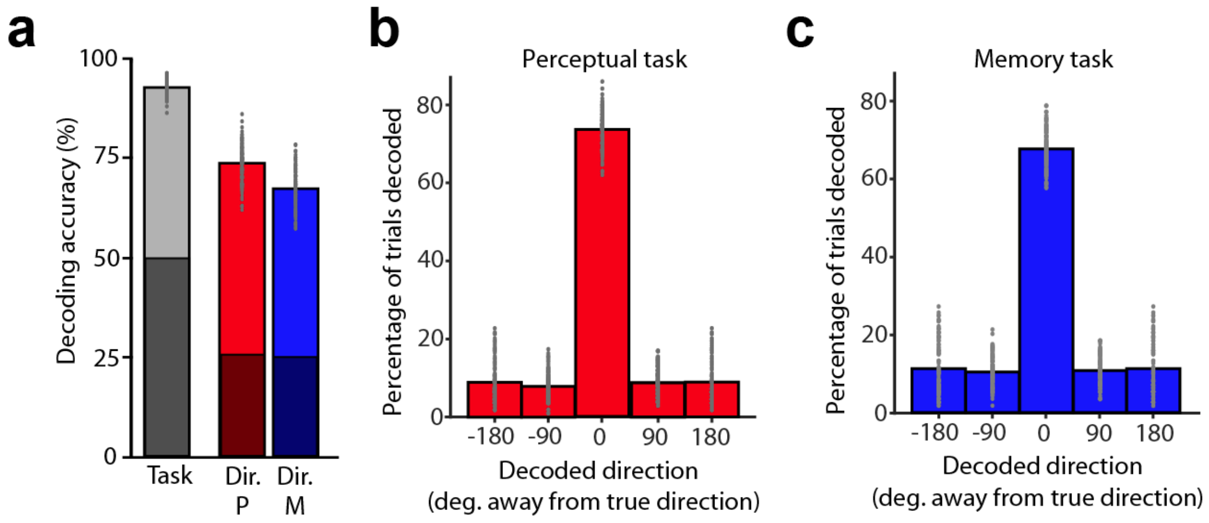


**Supplementary Figure 5. Decoding results obtained after randomly swapping direction labels between conditions.** (a) Percentage of trials classified as the correct task (gray) or the correct sample direction during the perceptual task (red) or memory task (blue). Conventions as in Fig. 4b. (b,c) Percentage of trials from the perceptual (b) and memory (c) tasks decoded as having a direction 0°, 90°, -90° or 180° away from their true direction. Conventions as in Fig. 6a,b.
